# Correction to: Selected abstracts from the 47th meeting of the Irish Endocrine Society Sligo, 24th & 25th November 2023

**DOI:** 10.1007/s11845-025-03907-6

**Published:** 2025-02-24

**Correction to: Irish Journal of Medical Science (1971 -) (2024) 193 (Suppl 5):S163–S212**

10.1007/s11845-024-03823-1

These abstracts were initially published as non-open access despite being intended for open access publication. This issue has now been resolved.

The original article has been corrected.

